# Temperature-dependent interchromophoric interaction in a fluorescent pyrene-based metal–organic framework†
†Electronic supplementary information (ESI) available. CCDC 1897075 and 1897076. For ESI and crystallographic data in CIF or other electronic format see DOI: 10.1039/c9sc01422e


**DOI:** 10.1039/c9sc01422e

**Published:** 2019-05-14

**Authors:** Andrzej Gładysiak, Tu N. Nguyen, Richard Bounds, Anna Zacharia, Grigorios Itskos, Jeffrey A. Reimer, Kyriakos C. Stylianou

**Affiliations:** a Laboratory of Molecular Simulation (LSMO) , Institut des Sciences et Ingénierie Chimiques (ISIC) , Ecole Polytechnique Fédérale de Lausanne (EPFL Valais) , Rue de l'Industrie 17 , 1951 Sion , Switzerland . Email: kyriakos.stylianou@epfl.ch; b Department of Chemical and Biomolecular Engineering , University of California , Berkeley 94720 , USA; c Experimental Condensed Matter Physics Laboratory , Department of Physics , University of Cyprus , Nicosia 1678 , Cyprus

## Abstract

Compounds exhibiting tuneable fluorescence emission upon heating or cooling are considered smart materials as their optical properties can be exquisitely controlled by adjusting the external temperature. Herein, we report such a material, which is a porous pyrene-based metal–organic framework with a chemical formula of [Mg_1.5_(HTBAPy)(H_2_O)_2_]·3DMF (H_4_TBAPy = 1,3,6,8-tetrakis(*p*-benzoic acid)pyrene), named **SION-7**. The bulk solid material of **SION-7** can display either monomer or excimer fluorescence emission due to the temperature-dependent extent of interchromophoric interactions between the HTBAPy^3–^ ligands within the framework. Consequently, the fluorescence emission colours gradually change from blue at low temperature (80 K) to yellow-green at high temperature (450 K). Interestingly, while kept in a relatively wide temperature range of 80–200 K, **SION-7** displays a structured monomer-like spectrum and its colour changes reversibly from deep to light blue. *Ex situ* variable-temperature (100–350 K) single-crystal X-ray diffractometry studies revealed the impact of solvent content on the optical properties of **SION-7**, and illustrated the correlation between the position of the phenylene groups of the HTBAPy^3–^ ligands at different temperatures and the interchromophoric interaction. Our study demonstrates a step forward towards the design of tuneable thermofluorochromic materials sought by optoelectronics.

## Abstract 2

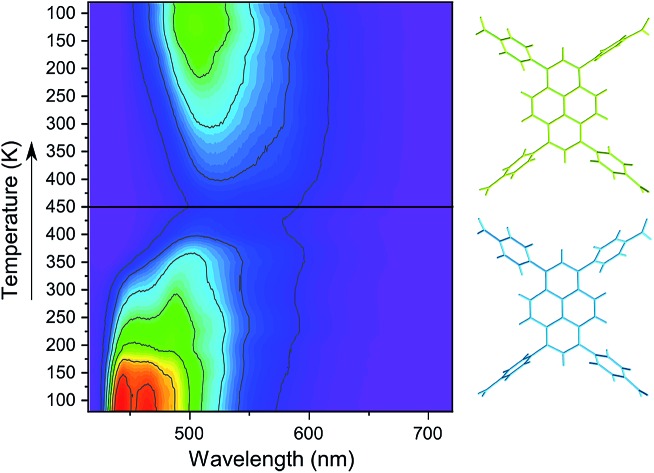
Variable temperature experiments revealed that the fluorescent emission colour of the pyrene-based **SION-7** changes from blue at 80 K to yellow-green at 450 K.

## Introduction

Interchromophoric interactions play a crucial role in the optical properties of materials that are assembled from multiple chromophores. For non-interacting chromophores, after photoexcitation, the excited electron can simply relax to the ground state *via* fluorescence and/or *via* non-radiative processes such as internal conversion and intersystem crossing. When the chromophores are coupled, *e.g.* when they are positioned in proximity to each other and with a suitable orientation, however, an excited chromophore can pair up with a nearby unexcited chromophore to form an excimer (excited dimer), which often displays emission that is markedly different than the one of the chromophore itself.^1^ When the components of the interacting chromophores are of different chemical identity, the entity is called exciplex (excited complex). Excimers and exciplexes have found numerous applications. They can be used to estimate the distance between biomolecules grafted with organic chromophores,^2^ while their application in chemical sensing or luminescent thermometry has also been widely studied.^3^ In addition, while embedded in a polymeric matrix, they can serve as wavelength switchable microlasers.^4^

Among fluorescent organic compounds, pyrene and its derivatives are canonical examples that display excimer fluorescence, which is often a structureless emission band in high-concentration solutions. When the solution is diluted, the structured emission band at lower wavelengths originating from the unassociated monomer becomes more dominant as the interaction between the pyrene chromophores is diminishing.^5^ This principle of interchromophoric interaction is also applied for pyrene and its derivatives in the solid state.^6^ Apparently, if this interaction can be finely controlled, materials with desired optical properties can be rationally designed. In fact, efforts in investigating the interchromophoric interaction in pyrene-based organic and inorganic solid materials have been noticed.^7^ For the latter, recent focus is on pyrene-based metal–organic frameworks (MOFs).^8^

MOFs are crystalline materials which often exhibit high porosity and structural tuneability,^9^ and can be synthesised in a wide range of topologies.^10^ They have found numerous applications, including methane storage,^11^ heterogeneous catalysis^12^ as well as temperature,^13^ pressure,^14^ and chemical sensing.^13^,^15^ Optical properties of MOFs, such as their UV/vis absorption, are known to be finely tuneable by external stimuli, *e.g.* chemical species,^16^ temperature^17^ or pressure.^18^ The fluorescence of MOFs has been equally intensely investigated.^19^ This arises from the fact that in many instances, the usefulness of MOF materials stems from their fluorescence emission, which in turn is heavily dependent on the interactions between the chromophores. Excimer emission of MOF materials due to interchromophoric interactions have been observed in several cases.3a,b,^20^ In particular, the extent of the interchromophoric interaction in several MOFs was found to be topology-dependent as each MOF displayed different concentration, distance, and mutual orientation of the ligands.7c,d However, it is still unclear how the interchromophoric interaction can be controlled within each porous material; therefore, this is still an attractive platform for further investigation, and is the topic of this work.

Herein, we report a porous MOF, named **SION-7**, based on Mg^II^ and H_4_TBAPy (1,3,6,8-tetrakis(*p*-benzoic acid)pyrene). The intensity and position of the fluorescence emission band of **SION-7** is temperature-dependent, with a structured emission spectrum characteristic for pyrene-derivative monomers at low temperature, which gradually transforms to a structureless red-shifted emission spectrum when the temperature is increased, indicating the presence of excimers due to interchromophoric interaction. *Ex situ* variable-temperature (VT) single-crystal X-ray diffractometry (SCXRD) studies were performed providing insights into the relationship between the structure and the temperature-dependent interaction between the pyrene moieties, and will be discussed in detail.

## Experimental section

### Procedures and materials

All manipulations were performed under aerobic conditions using chemicals and solvents as received without further purification. The compound H_4_TBAPy was synthesised using a previously reported procedure.8*a*

### Synthesis of **SION-7**

With the chemical formula of [Mg_1.5_(HTBAPy)(H_2_O)_2_]·3DMF, **SION-7** was synthesised from the reaction of 10.0 mg (0.0390 mmol) of Mg(NO_3_)_2_·6H_2_O and 10.0 mg (0.0146 mmol) of H_4_TBAPy in a mixture composed of 5.0 mL of *N*,*N*-dimethylformamide (DMF), 1.0 mL of H_2_O and 80 μL of HCl (Techn., 32%) held at 393 K for 72 hours. Block-shape single crystals of **SION-7** suitable for SCXRD analysis were obtained in a 47.8% yield (6.8 mg). The activated sample, **SION-7a**, was prepared by heating the dried powdered sample of **SION-7** at 403 K under dynamic vacuum for 8 hours.

### Single-crystal X-ray diffraction (SCXRD)

A high-quality single crystal of **SION-7** was isolated from the mother liquor, and mounted onto the PILATUS@SNBL diffractometer at the BM01 beamline (European Synchrotron Radiation Facility, Grenoble, France).^21^ The crystal was kept at 100 K, probed with X-rays, and the intensities of Bragg reflections were recorded with the PILATUS2M detector. Raw data were processed with CrysAlisPro (v. 1.171.38.43) program suite,^22^ and the empirical absorption correction was performed using spherical harmonics, implemented in SCALE3 ABSPACK scaling algorithm. Crystal structure was solved with the SHELXT structure solution program using Intrinsic Phasing,^23^ and refined with the SHELXL refinement package using least-squares minimisation,^24^ implemented in the Olex2 program suite.^25^ Structure simplification and net classification was performed using the TOPOS Pro program suite.^26^ Crystal structure-derived pore volume as well as contribution of the disordered solvent molecules found in the structural voids to the measured structure factors were quantified with the solvent mask procedure implemented in Olex2.^27^ The integrated electron density found in the pores was interpreted in terms of number of DMF molecules by comparison to the electron count of the latter (C_3_H_7_NO, 40 e^–^).

Variable-temperature (VT) SCXRD measurements were performed by modifying the abovementioned procedure. The temperature was controlled with a Cryostream 700+ nitrogen blower. Full sphere of reflections was recorded at each temperature point, and the corresponding crystal structures were further solved and refined. Eventually, crystal structure of **SION-7** was determined at 100, 150, 200, 250, 270, 293, 310, 330, and 350 K on heating and subsequent cooling.

### Other characterisation techniques

Powder X-ray diffraction (PXRD) patterns were recorded using synchrotron radiation at the BM31 beamline (ESRF, Grenoble, France). In the *in situ* VT PXRD experiment bulk powder of the as-synthesised **SION-7** was packed into a glass capillary and heated at a rate of 3 K min^–1^ from room temperature to 600 K. The Le Bail fit of the pattern recorded at room temperature was performed with the FullProf program suite.^28^

Thermogravimetric analysis (TGA) was performed with the TGA Q500 instrument on a sample heated at a constant rate of 5 K min^–1^ with air acting as carrier gas.

Gravimetric gas sorption measurements were performed using the Intelligent Gravimetric Analyzer Instrument (IGA) from Hiden.

^13^C CP-MAS solid-state NMR spectra were recorded at a 700 MHz spectrometer with a rotor spinning frequency of 8 kHz. The density functional theory (DFT)-based code CASTEP was used to calculate the predicted chemical shifts.

Fourier-transform infrared (FT-IR) spectra were recorded on a PerkinElmer Spectrum Two FT-IR spectrometer.

Diffuse reflectance UV/vis spectra were recorded on a PerkinElmer Lambda 950 S spectrometer.

VT fluorescence spectra were recorded in the 80–450 K temperature range in steps of 10 K (80–200 K range) or 25 K (200–450 K range), as well as in the 80–200 K temperature range in steps of 10 K, covering the range of wavelengths of 415–720 nm, using the excitation wavelength of 405 nm. In addition, the fluorescence spectra were expressed in the RGB coordinates using the Commission Internationale de l'Eclairage (CIE)-1931 standard. Fluorescence decays monitoring the peak emission were fitted with double-exponentials yielding weighted average lifetimes.

## Results and discussion

### Synthesis and crystal structure analysis

The synthesis of **SION-7** was performed under solvothermal reaction conditions. Mg^2+^ was chosen to form the MOF as it is diamagnetic and does not cause the quenching of fluorescence often observed with paramagnetic metal ions. The presence of a small amount of HCl is critical for the reaction and it is believed to act as the reaction modulator that facilitates crystal growth by adjusting the reaction kinetics. The molar ratios of the reactants were screened to obtain the phase-pure product. The synthesis procedure is slightly different to that previously reported,^29^ therefore, the formulae of the resultant MOFs differ.

**Fig. 1:**
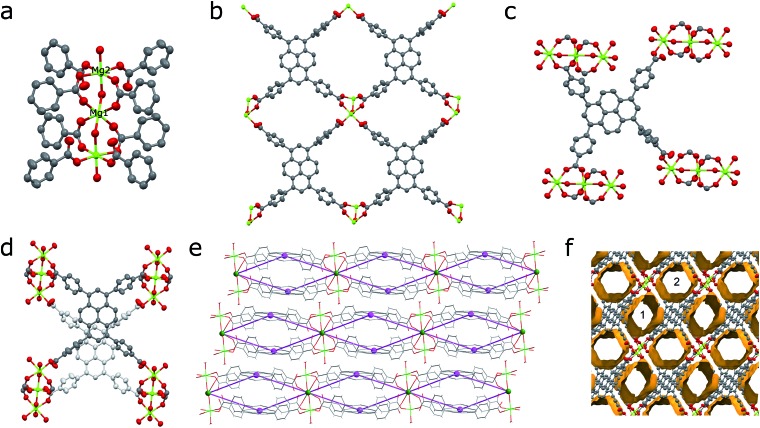
(a) Centrosymmetrical Mg_3_-cluster constituting the structure of **SION-7**. Mg1, positioned in its middle, sits on an inversion centre, while Mg2 is on a general position. (b) Top part of a 2-dimensional layer constructed from HTBAPy^3–^ ligands joined together by Mg_3_-clusters. (c) Coordination environment of an HTBAPy^3–^ ligand with η^1^:η^1^-bridging and η^1^-coordination modes. (d) A pair of HTBAPy^3–^ ligands linked by a Mg_3_-cluster viewed parallel to the pyrene cores (the ligand positioned in the bottom is shown in pale grey). Offset of pyrene cores is noticeable. (e) 2-Dimensional layers viewed along the [11[combining macron]0] direction overlaid with the topological scheme of the underlying **4,8L15** net. (f) The system of structural voids, including pore 1 and pore 2, viewed along the *a*-axis. Colour code: C, black; O, red; Mg, light green; 4-c node, purple; 8-c node, green. H atoms are omitted for clarity.

The structure of **SION-7** was revealed from the SCXRD measurement. **SION-7** crystallises in the triclinic space group *P*1[combining macron]. The asymmetric unit comprises one partially protonated HTBAPy^3–^ ligand, two Mg atoms (one of which sits on a special position with 0.5-occupancy), and two coordinated H_2_O molecules (Fig. S1†). **SION-7** is a 2-dimensional structure in which Mg–O trinuclear clusters (Fig. 1a) link pairs of mutually parallel HTBAPy^3–^ ligands into infinite layers (Fig. 1b). In the third dimension, the clusters form supramolecular chains along the *a*-axis held together by hydrogen bonds (Fig. S2†). Within each of these clusters, Mg1 assumes the central position (it is localised on an inversion centre), Mg2 is present in form of two symmetrical equivalents, and both Mg1 and Mg2 adopt an octahedral coordination geometry with the coordination number 6 (Fig. 1a). Such clusters are not uncommon in the coordination chemistry of magnesium.^30^ Four HTBAPy^3–^ positioned in the middle of the Mg_3_-cluster use both their carboxylate O-atoms to bind to Mg1 and Mg2 through the η^1^:η^1^-bridging mode, while the other four HTBAPy^3–^ ligands are η^1^-coordinated to Mg2 through a single carboxylate O-atom (Fig. 1c). Analogous types of positions are assumed by coordinated H_2_O molecules, with two of them in the middle of the Mg_3_-cluster being η^1^:η^1^-bridging and two others at its extremities being η^1^-coordinated. In addition, HTBAPy^3–^ ligands η^1^-coordinated to Mg2 are bound with strong hydrogen bonds to the bridging H_2_O molecules (Fig. S3†). Pairs of HTBAPy^3–^ ligands are linked covalently by coordination bonds (Fig. 1d); the 2D layers, in turn, are stacked on the top of one another with non-covalent (H-bonds, π–π stacking and van der Waals) interactions. The layered structure of **SION-7** can be topologically interpreted considering the Mg_3_-clusters as 8-coordinated nodes, and HTBAPy^3–^ ligands as 4-coordinated nodes. This interpretation leads in the **4,8L15** net described by the Schläfli symbol of (4^20^·6^8^)(4^6^)_2_ (Fig. 1e and S4†).^31^ The structure of **SION-7** contains infinitely propagating structural voids, and two distinct pores, pore 1 and pore 2 (Fig. 1f), account for the total of 41.1% of the unit cell's volume and accommodate three heavily disordered DMF molecules.

### Thermal stability and phase transitions

**Fig. 2:**
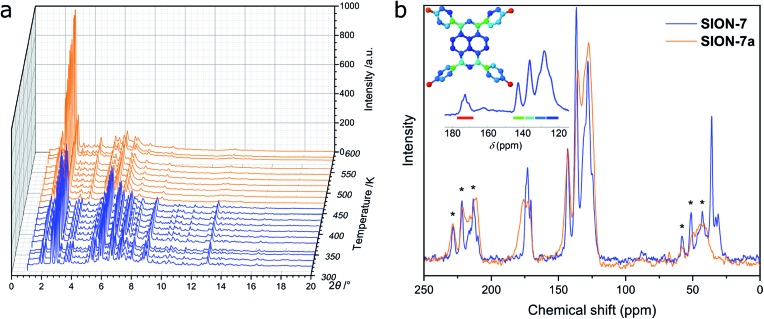
(a) VT PXRD patterns recorded from **SION-7** on heating. Significant changes in the positions and intensities of the Bragg peaks at 456 K marking the phase transition towards **SION-7a** are clearly noticeable. (b) ^13^C CP-MAS solid-state NMR spectrum of **SION-7a** compared to that of **SION-7**. Asterisks refer to spinning sidebands. Peak assignment: 170–180 ppm: carbonyl C of HTBAPy^3–^, 161.8 ppm: carbonyl C of DMF,^32^ 120–145 ppm: aromatic C (pyrene core and phenylene rings) of HTBAPy^3–^, 30.6 and 35.7 ppm: alkyl C of DMF.^32^ The inset illustrates the DFT-calculated resonances and their assignment to the structure.

Le Bail fit confirmed the phase purity of **SION-7** (Fig. S5†). The structure of **SION-7** is retained on heating up to 456 K, as inferred from *in situ* VT PXRD measurements (Fig. 2a). At this temperature a phase transition occurs towards **SION-7a** endowed with a markedly different powder pattern. Significant change of periodicity shown by PXRD indicates a structural rearrangement taking place upon the phase transition. The phase transition temperature of 456 K is also registered by the TGA: this is the point up to which all three guest DMF molecules are released in a gradual way (Fig. S6†). Therefore, we reason that **SION-7a** is the activated form of **SION-7**, *i.e.* the one of the same chemical composition, but with solvent-free pores. This is further confirmed by the gravimetric sorption measurements, which show that **SION-7a** is porous to N_2_ at 77 K and 1 bar, and CH_4_ (Fig. S7†), and its BET surface area amounts to 580 m^2^ g^–1^. The structure-derived pore volume (0.30 cm^3^ g^–1^) is consistent with the pore volume obtained from the N_2_-adsorption isotherm (0.22 cm^3^ g^–1^). Additionally, the ^13^C CP-MAS solid-state NMR spectrum of the as-made **SION-7**, successfully predicted with the DFT-based CASTEP code, displays the same resonances as the spectrum of the activated **SION-7a** does (Fig. 2b), and the FT-IR spectra of **SION-7** and **SION-7a** are nearly identical (Fig. S8†), hence upon activation the chemical identity of the material is retained. This activation, however, is irreversible as submersion of **SION-7a** in liquid DMF does not lead to the regeneration of **SION-7** (confirmed by PXRD). **SION-7a** is stable on heating up to around 700 K, when the framework decomposition starts (TGA, Fig. S6†). *In situ* activation at elevated temperatures under vacuum leads to the breakdown of single crystals of **SION-7** (Fig. S9†), thus preventing us from the study of **SION-7a** with SCXRD.

### Optical properties

Bulk **SION-7** powder has a pale yellow colour, which manifests itself in a diffuse reflectance UV/vis spectrum as a broad peak centred at 413 nm. Upon activation, the colour of **SION-7a** changes to deep yellow, which is also reflected by a slight shift of the absorption band towards a maximum at 434 nm (Fig. S10†). By exciting **SION-7** near its absorption maximum at rt. results in a fluorescence emission with a broad peak centred at around 500 nm and with the calculated quantum yield of 1.59(2)%. The shift observed in the UV/vis diffuse reflectance spectra of **SION-7** and **SION-7a** as well as the widely studied fluorescent properties of the pyrene molecule and its derivatives prompted us to thoroughly investigate the emission of **SION-7** using variable-temperature fluorescence spectroscopy.15c,^33^

### Temperature-dependent interchromophoric interaction

**Fig. 3:**
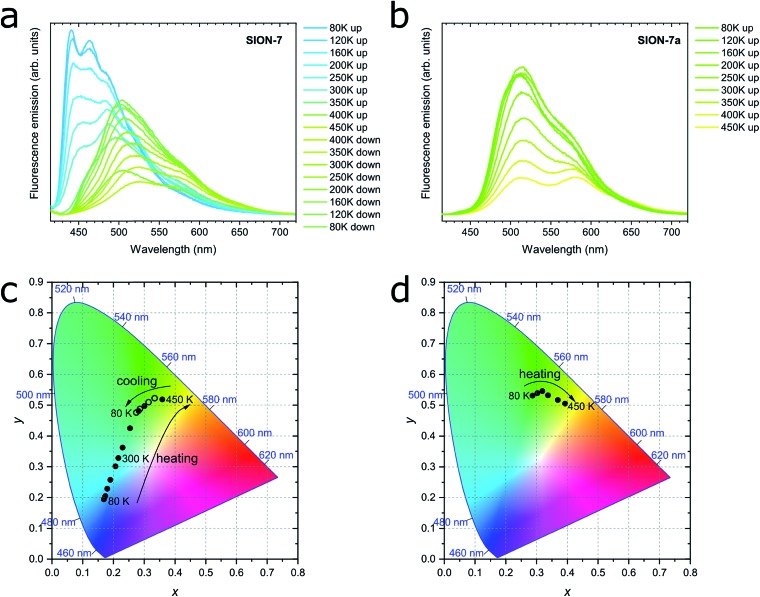
(a) Fluorescence spectra of **SION-7** measured in the 80–450 K temperature range on heating and cooling. (b) Fluorescence spectra of **SION-7a** measured in the 80–450 K temperature range on heating. In both panels the colours of the curves represent the perception of a given spectrum by human colour vision, as determined with the CIE-1931 standard. *λ*_ex_ = 405 nm. (c) CIE-1931 chromaticity diagram displaying the colour coordinates (*x*, *y*) of the fluorescence from **SION-7** during heating (filled circles) and cooling (empty circles) in the 80–450 K temperature range. (d) CIE-1931 chromaticity diagram of the fluorescence from **SION-7a** during heating from 80 to 450 K.

Solid-state fluorescence emission spectra of **SION-7** measured in the 80–450 K temperature range on heating and cooling are presented in Fig. 3a. At 80 K, **SION-7** exhibits a broad fluorescence spectrum including distinctly structured bands at 440 nm and 465 nm, characteristic for the π–π* transitions of pyrene-derived monomers.^5^ Upon heating, this emission decreases in intensity and the structureless emission bands characteristic of the excimer become more dominant. At 450 K, the excimer emission with the peak at 525 nm can be observed. A similar transition was reported in thin films of high-temperature polymorph of pyrene,^34^ but not in a bulk solid material. This result clearly demonstrates the varying degree of interchromophoric interaction within **SION-7** as a function of temperature. On subsequent cooling, the monomer spectrum is not recovered, but instead the excimer emission enhances its intensity and blue-shifts its peak to 505 nm at 80 K (Fig. 3a). CIE-1931 chromaticity diagram of the **SION-7** fluorescence shows the gradual transition of the emission colour from blue at low temperatures to yellow-green at higher temperatures (Fig. 3c and S11†). Although MOFs responding to external pressure^35^ and chemical species^36^ alter the colour of their fluorescence to a similarly strong extent, such drastic changes occurring in MOFs as a function of temperature are relatively rare.^37^ Further heating and cooling of the material gives a very similar emission and intensity profile as in the first thermal sequence (Fig. S12a, c and e†), in which the fluorescence intensity always decreases upon heating, but is brought back to the initial values on cooling (Fig. S12b, d and f†). This is expected since at higher temperatures more vibrational levels are available, and hence more non-radiative decay processes can occur. Emission lifetimes of **SION-7** at different temperatures within the first thermal sequence are summarised in Table 1. It is apparent that upon heating, the increased population of excimers leads to the lengthening of the fluorescence dynamics. This observation is in agreement with a previous report in which the excimer formation in pyrene-based MOFs enhances the lifetime of the emissive excited states as compared to the free linker.7*c*

**Table 1:** Average fluorescence lifetimes of **SION-7** at different temperatures upon heating and cooling

Temperature (K)	Average lifetime (ns)	
	Heating, 80–450 K	Cooling, 450–80 K
100	2.08	4.55
200	2.31	4.23
450	2.72	2.72

Due to the phase transition observed when **SION-7** is activated and transforms into **SION-7a**, the fluorescence emission of **SION-7a** was also investigated. Like **SION-7** in the second and third thermal sequences, **SION-7a** exhibits a similar featureless excimer emission in the entire 80–450 K temperature range, and the emission profiles are reversible for at least three cycles of heating and cooling (Fig. 3b and S13†). The colour of the fluorescence changes from yellow-green back and forth to yellow (Fig. 3d and S14†). The immersion of **SION-7a** in DMF does not lead to the recovery of the monomer emission of the resultant material (Fig. S15†).

**Fig. 4:**
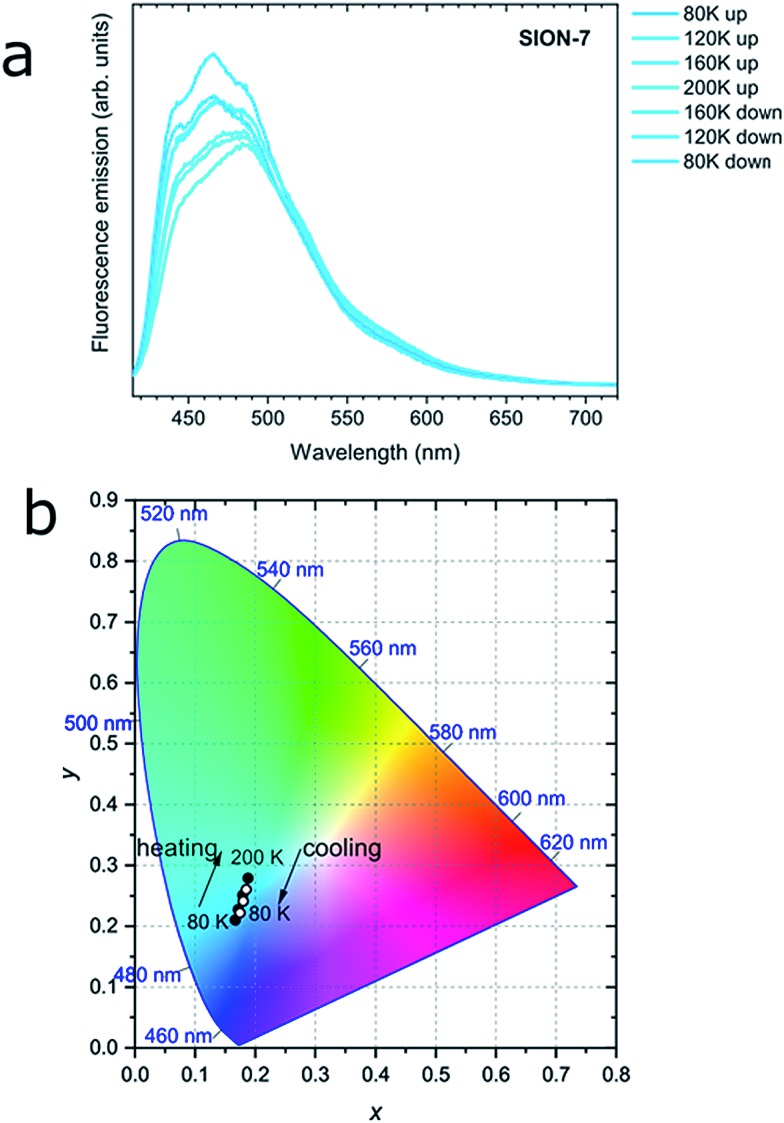
(a) Fluorescence spectra of **SION-7** measured in the 80–200 K temperature range on heating and cooling. (b) CIE-1931 chromaticity diagram of the fluorescence from **SION-7** during heating (filled circles) and cooling (empty circles) in the 80–200 K temperature range.

Interestingly, while kept in a relatively wide temperature range of 80–200 K, as-made **SION-7** displays structured monomer-like spectrum, which red-shifts and decreases in intensity when the temperature is increased, and blue-shifts and increases in intensity when the temperature is decreased (Fig. 4a and S16†). Its colour changes reversibly from deep to light blue, but never surpasses the left bottom part of the CIE-1931 chromaticity diagram (Fig. 4b and S17†). Therefore, be keeping the material in a low-temperature range, monomer emission from **SION-7** can be retained.

**Fig. 5:**
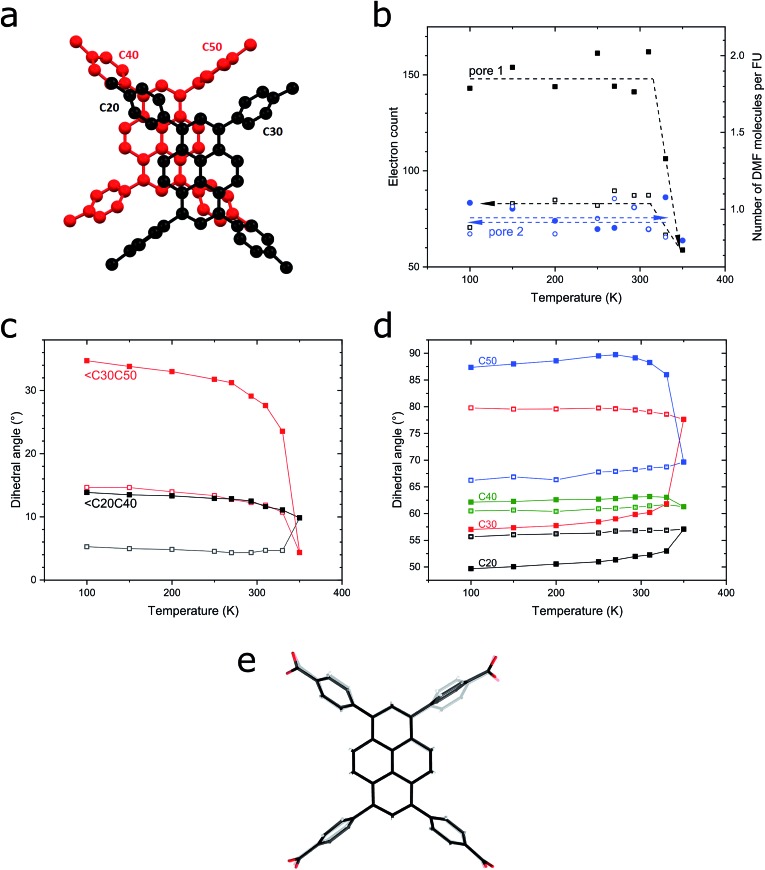
(a) A pair of HTBAPy^3–^ ligands originating from two neighbouring 2-dimensional layers, forming an excimer. Phenylene rings are captioned. (b) Content of pore 1 and pore 2 of **SION-7** expressed as electron count (left vertical axis) and the corresponding number of DMF molecules per formula unit (right vertical axis). (c) Dihedral angles between the phenylene rings originating from the neighbouring HTBAPy^3–^ within the excimer plotted as a function of temperature. (d) Dihedral angles between the pyrene core and the phenylene rings within each HTBAPy^3–^ ligand. Full and empty symbols denote the heating and cooling regimes, respectively. (e) Superposition of the images of HTBAPy^3–^ derived from the structures at 100 K (opaque wireframe) and 350 K (bold wireframe).

To elucidate the excimer formation in **SION-7**, VT SCXRD measurements were performed. As previously described, the structure of **SION-7** consists of 2-dimensional layers of pairs of HTBAPy^3–^ ligands joined together by Mg_3_-clusters non-covalently stacked on the top of one another (Fig. 1c). Interestingly, the pyrene core planes spacing within the Mg_3_-bound layers (4.752 Å in the initial crystal structure measured at 100 K) is greater than the spacing between planes of pyrene cores originating from two subsequent layers (3.561 Å). Since also the offset between the succeeding pyrene cores is greater within the layers than in between of them (Fig. 5a and S18a, b†), we reason that the pyrene cores originating from two consecutive 2-dimensional layers interact with each other to give the excimer fluorescence. This spacing increases with temperature (by 0.24 Å in the 100–400 K temperature range; Fig. S18c†), and amounts to slightly higher values than the sum of the van der Waals radii of C (3.54 Å).^38^ However, this observation only explains the excimer emission in **SION-7a** and in **SION-7** during the second and third thermal sequence, but does not explain the monomer emission exhibited by the as-made **SION-7**. The clarification comes from the analysis of the pore content as a function of temperature. As aforementioned, there are two types of pores within the structure of **SION-7**, pore 1 and pore 2. Pore 1, situated at *x*, 0, 0.5, is at the initial stage at 100 K filled with *ca.* 2 DMF molecules, while pore 2, with the fractional coordinates of *x*, 0.5, 0, encloses *ca.* 1 DMF molecule. As the temperature is increased, the total pore content changes to a limited extent up to 330 K, and at this temperature a dramatic decrease in electron count inside pore 1 takes place (Fig. 5b). Above this point and on subsequent cooling, the content of this pore stays virtually invariant as it now comprises *ca.* 1 molecule of DMF. Simultaneously, the content of pore 2 amounts to *ca.* 1 DMF molecule throughout the entire thermal cycle (Fig. 5b), and the pore content analysis is consistent with the TGA result (Fig. S5†). It is reasonable that extra DMF molecules in pore 1 impede the pyrene–pyrene interactions hindering the formation of an excimer, which can be formed and show up in the emission spectra solely once the guest solvent molecules are released. Therefore, fluorescence of **SION-7** is a physical phenomenon which is strongly dependent on the presence of the guest solvents, in analogy to spin crossover in Fe_2_(azpy)_4_(NCS)_4_,^39^ and compressibility in Zn(niba)_2_(OH)_2_.^40^ Another structural feature that explains the excimer formation is the level of extended π–π conjugation of the aromatic rings. Within each HTBAPy^3–^ excimer, the pyrene cores are parallel to each other, while the phenylene rings attached to them are positioned in a much less regular manner. As the temperature is increased, the values of dihedral angles between the phenylene rings originating from the neighbouring HTBAPy^3–^ within the excimer (captioned as C20–C40 and C30–C50 in Fig. 5a) decrease: ∠C20C40 drops from 13.86° at 100 K at the beginning of the thermal cycle to 9.85° at 350 K, while the corresponding initial and final values for ∠C30C50 are 34.72° and 4.37°, respectively (Fig. 5c). This implies that the phenylene rings effectively become more parallel towards each other (a dihedral angle of 0° would signify a perfect parallelism), thus the interaction between them is more favourable. Moreover, the dihedral angles between the pyrene core and the phenylene rings within each HTBAPy^3–^ ligand change in a multidirectional way with temperature (the angles C20–pyrene and C30–pyrene increase, C40–pyrene stays virtually invariant, while C50–pyrene decreases, Fig. 5d), which may explain why slight changes in the fluorescence shift and intensity are still observed even after **SION-7** undergoes the transition towards the excimer state. Superimposing the images of HTBAPy^3–^ derived from the structures at 100 K and 305 K emphasises the multidirectional way of phenylene–pyrene dihedral angle changes in the discussed temperature range (Fig. 5e).

## Conclusions

**SION-7**, a MOF synthesised from the self-assembly of Mg^2+^ and H_4_TBAPy, was found to be a platform suitable for an in-depth study of the interactions between pyrene derivative chromophores incorporated into the metal–organic framework. Noteworthy, it was found that the interchromophoric interactions in a pyrene-based MOF do not depend solely on the framework topology and the linker concentration, but can also be tuned by external stimuli. In particular, temperature was shown to dramatically change the mutual orientation of HTBAPy^3–^ ligands (enhanced parallelism of the phenylene rings originating form two neighbouring layers of HTBAPy^3–^ ligands plus multidirectional change of the phenylene–pyrene dihedral angles at elevated temperatures) and the pore content of **SION-7** (at 100 K pore 1 is filled with approx. 2 DMF, and pore 2 with approx. 1 such molecule; upon heating up to 350 K, the content of these pores changes to approx. 1 and 1 DMF molecule, respectively), which entails crucial changes in the fluorescence emission (a structured monomer emission at low temperatures transforms to a structureless excimer emission at high temperatures). Another important conclusion of this work is that the presence of simple guest molecules such as solvents plays an important role in the fluorescence emission of pyrene-based MOFs, and shall not be overlooked in future studies. Our study opens a new dimension for controlling the optical properties of porous materials since the colour of their emission can be regulated without the need of introducing additional chemical species. When integrated to a substrate, *e.g.* in form of a thin film, **SION-7** could potentially serve as a temperature sensor for cryogenic environments; when temperatures higher than cryogenic were attained, such a sensor would dramatically change its colour.

## Conflicts of interest

There are no conflicts to declare.

## Supplementary Material

Supplementary information

Crystal structure data
