# Supplementary material for: Temperature-dependent interchromophoric interaction in a fluorescent pyrene-based metal–organic framework
Source: Chem Sci. 2019 May 14;10(24):6140–8. doi: 10.1039/c9sc01422e (PMC6585595; doi:10.1039/c9sc01422e)
Supplement: Supplementary file 1 [file SC-010-C9SC01422E-s001.pdf]

Electronic Supplementary Material (ESI) for Chemical Science.
This journal is © The Royal Society of Chemistry 2019

Temperature-dependent Interchromophoric Interaction in a Photoluminescent Pyrene-based Metal–organic Framework

*Andrzej Gładysiak, Tu N. Nguyen, Richard Bounds, Anna Zacharia, Grigorios Itskos, Jeffrey A. Reimer, Kyriakos C. Stylianou**

Supporting information

Table 1. Crystal data and structure refinement for **SION-7** at 100 K.

Identification code	SION_T100
Empirical formula	$C_{106}H_{96}Mg_3O_{26}N_6$
Formula weight	1942.81
Temperature/K	100.0
Crystal system	triclinic
Space group	P-1
a/Å	11.2723(4)
b/Å	14.4121(5)
c/Å	15.9112(5)
α/°	81.410(3)
β/°	77.802(3)
γ/°	77.284(3)
Volume/Å^3	2450.19(15)
Z	1
ρ_{calc}g/cm^3	1.317
μ/mm^{-1}	0.112
F(000)	1018.0
Crystal size/mm^3	0.18 × 0.11 × 0.08
Radiation	synchrotron (λ = 0.7142)
2Θ range for data collection/°	4.154 to 63.946
Index ranges	$-16 \leq h \leq 16$, $-18 \leq k \leq 18$, $-23 \leq l \leq 23$
Reflections collected	18468
Independent reflections	9526 [R_{int} = 0.0176, R_{sigma} = 0.0311]
Data/restraints/parameters	9526/6/503
Goodness-of-fit on F^2	1.086
Final R indexes [I>=2σ (I)]	R_1 = 0.0701, wR_2 = 0.2070
Final R indexes [all data]	R_1 = 0.0726, wR_2 = 0.2105
Largest diff. peak/hole / e Å^{-3}	0.50/-0.48
CCDC	1897075

Table 2. Crystal data and structure refinement for **SION-7** at 350 K.

Identification code	SION_T350
Empirical formula	$C_{96.38}H_{67.56}Mg_{3}N_{2.79}O_{22.79}$
Formula weight	1702.28
Temperature/K	350
Crystal system	triclinic
Space group	P-1
a/Å	11.4034(5)
b/Å	14.6274(5)
c/Å	16.1091(7)
α/°	80.439(3)
β/°	77.037(4)
γ/°	75.856(3)
Volume/Å^3	2521.50(19)
Z	1
ρ_{calc}g/cm^3	1.121
μ/mm^{-1}	0.097
F(000)	884.0
Crystal size/mm^3	0.18 × 0.11 × 0.08
Radiation	synchrotron (λ = 0.7142)
2Θ range for data collection/°	3.772 to 63.59
Index ranges	$-16 \le h \le 16$, $-18 \le k \le 18$, $-23 \le l \le 23$
Reflections collected	18816
Independent reflections	9688 [R_{int} = 0.0255, R_{sigma} = 0.0507]
Data/restraints/parameters	9688/6/503
Goodness-of-fit on F^2	1.238
Final R indexes [I>=2σ (I)]	R_1 = 0.1059, wR_2 = 0.3011
Final R indexes [all data]	R_1 = 0.1120, wR_2 = 0.3093
Largest diff. peak/hole / e Å^{-3}	0.67/-0.43
CCDC	1897076

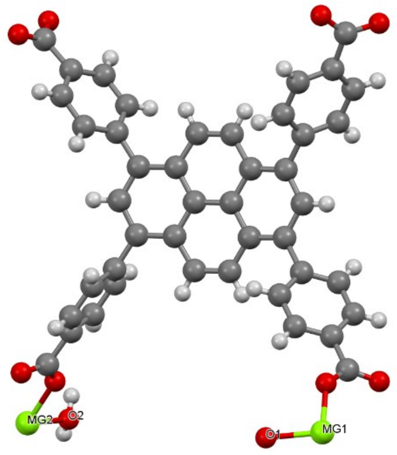

Fig. S1. Symmetrically independent part of the unit cell of **SION-7**. Apart from one $HTBAPy^{3-}$ ligand this comprises two Mg-atoms (of which Mg1 sits on a special position with 0.5-occupancy), and O1 and O2 oxygen atoms belonging to two coordinated H_2O molecules. Some H-atoms could not be localised.

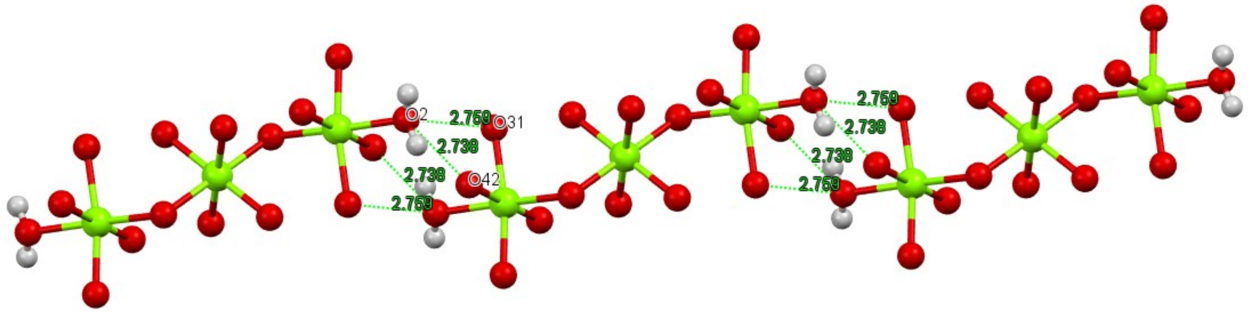

Fig. S2. A supramolecular chain formed by the extension of Mg_3-clusters along the *a*-axis by means of the hydrogen bonds O2···O31 (2.759 Å) and O2···O42 (2.738 Å).

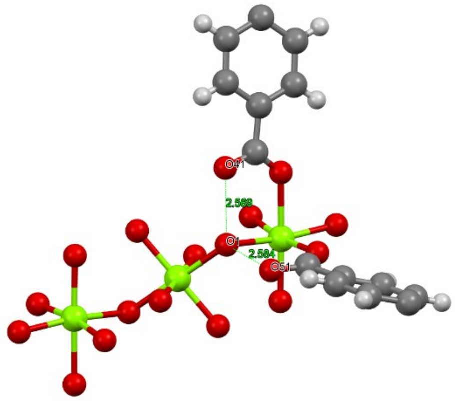

Fig. S3. Chemical environment of the O1 bridging atom within the Mg_3-cluster; numbers refer to the lengths (expressed in Å) of the corresponding hydrogen bonds. It is plausible that the H atom of the partially protonated $HTBAPy^{3-}$ ligand is disordered and shared by two protonated carboxylate groups bound with hydrogen bonds to the bridging H_2O molecule.

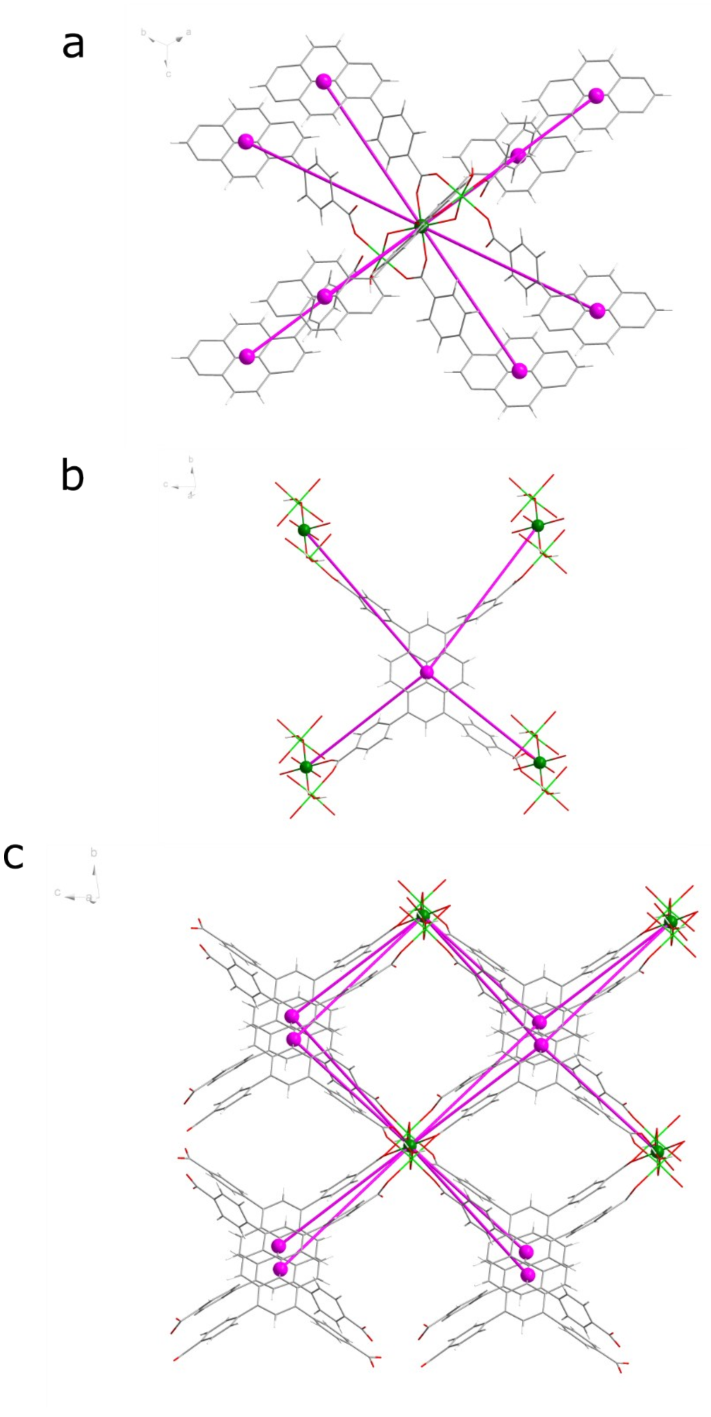

Fig. S4. SION-7 topologically interpreted as a 4,8-c net. (a) Mg_3-cluster (green sphere) interpreted as an 8-c node. (b) $HTBAPy^{3-}$ (purple sphere) interpreted as a 4-c node. (c) Fragment of the $(4^{20}.6^{8})(4^{6})_2$ underlying net viewed approximately along the *a*-axis.

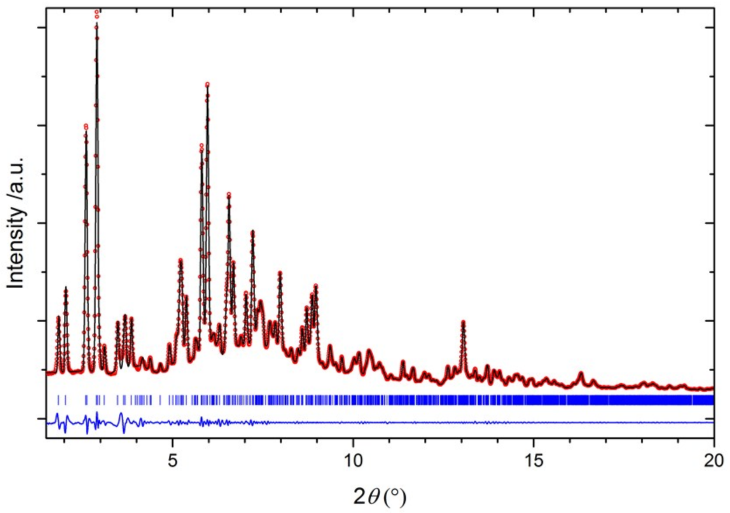

Fig. S5. Le Bail refinement of the **SION-7** PXRD pattern recorded with synchrotron radiation at rt. (space group $P\bar{1}$: R_p = 4.01%, R_{wp} = 4.26%; a = 11.3514(2) Å, b = 14.4851(4) Å, c = 15.9534(4) Å, α = 81.2688(14)°, β = 77.8212(19)°, γ = 76.5933(13)°; λ = 0.50084 Å). The red dots represent the experimental data, the black plots show the refined Le Bail profiles, while the blue plots represent the difference between them. Reflection positions are marked in blue.

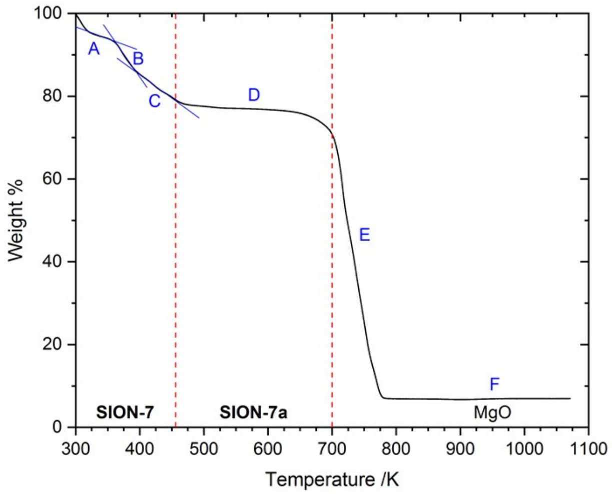

Fig. S6. TGA of **SION-7**. Steps A (up to 360 K, –6.6 wt%), B (360–395 K, –7.8 wt%) and C (395–456 K, –7.6 wt%) can each be approximated to 7.7 wt% loss of the framework, which, based on the formula, corresponds to the mass of 1 DMF molecule. Subsequent phase transitions at 456 K and 700 K are marked with dashed red lines. At 780 K, the remaining 6.8 wt% correspond to the entirely mineralised magnesium oxide residue (6.2 wt%). Since this analysis predicts a full desolvation of **SION-7**, no ions are expected to reside within the pores. Therefore, it is inferred that the singly protonated form of the ligand, $HTBAPy^{3-}$, constitutes the framework of **SION-7**.

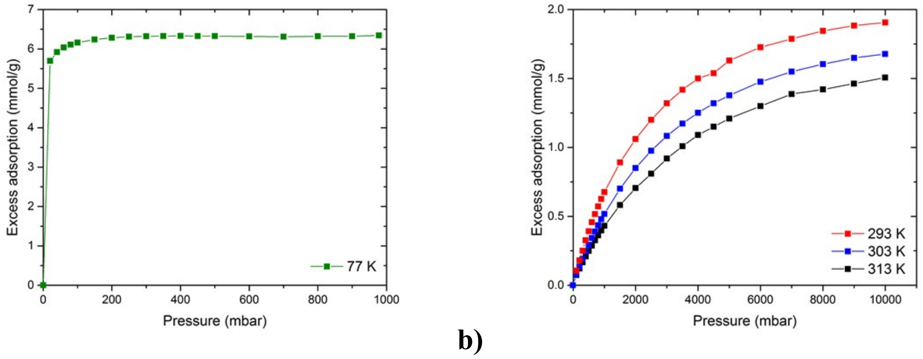

a) b)

Fig. S7. (a) N_2-adsorption type-I isotherm of **SION-7a** recorded at 77 K. Activation conditions: heating at 403 K under dynamic vacuum for 8 hours. The recorded weight loss (25.1%) is consistent with the weight loss derived from the TGA (24.3 wt%) and from the formula (26.3 wt%). (b) CH_4-adsorption type-I isotherms of **SION-7a** recorded at 293 K, 303 K, and 313 K.

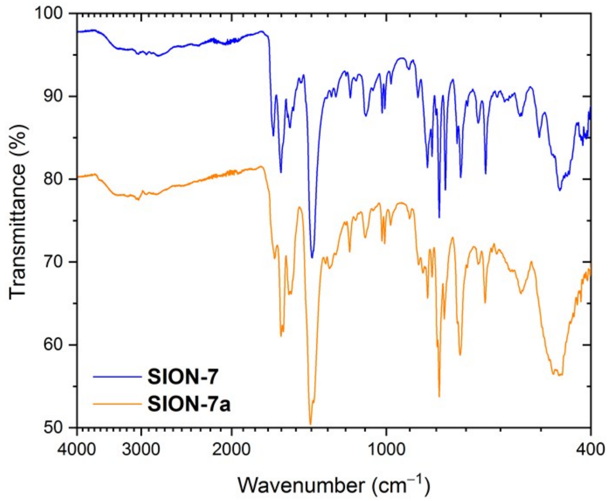

Fig. S8. FT-IR spectra of **SION-7** (blue plots) compared to those of **SION-7a** (orange plots). Occurrence of the same bands in the spectra indicate the presence of identical functional groups in the respective solid materials. The spectrum of **SION-7a** is offset by –15%.

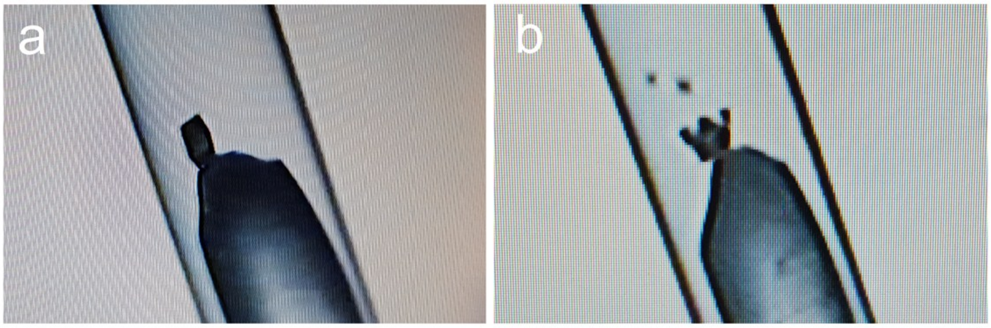

Fig. S9. Optical photographs of a single crystal of **SION-7** enclosed in an environment cell (a), and subjected to the vacuum of 10^{-3} mbar and the temperature above 350 K (b). As the single crystal shatters, the study of **SION-7a** with SCXRD proved impossible.

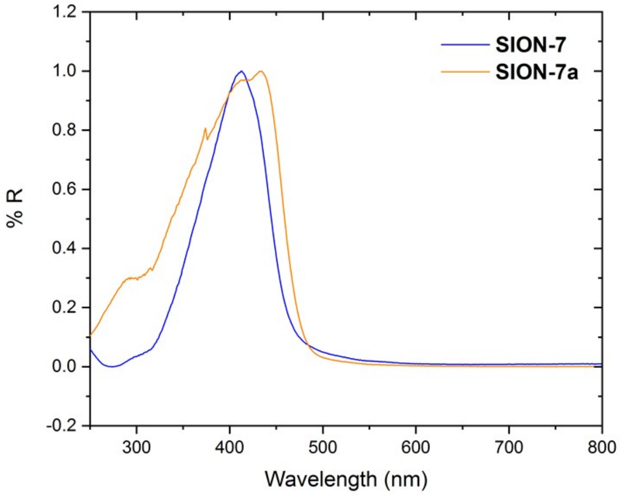

Fig. S10. Diffuse reflectance UV/vis spectra of **SION-7** and **SION-7a**. The spectra have been normalised for an easier comparison.

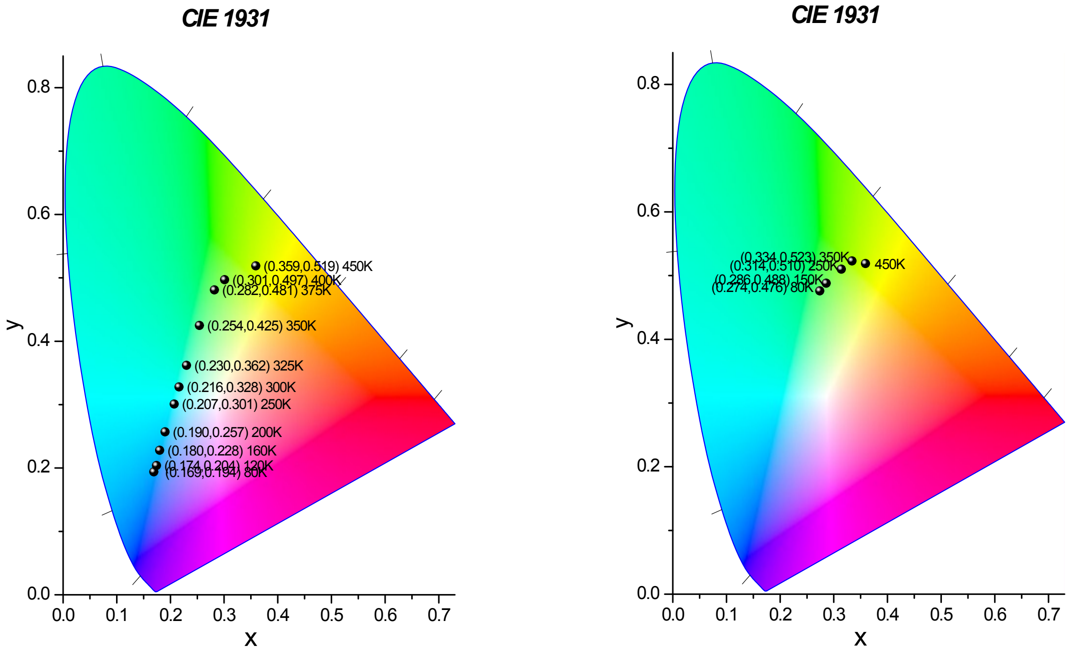

a) **b)**

Fig. S11. CIE-1931 chromacity diagram of the fluorescence from **SION-7** (a) on heating in the 80–450 K temperature range, and (b) on cooling in the 450–80 K temperature range.

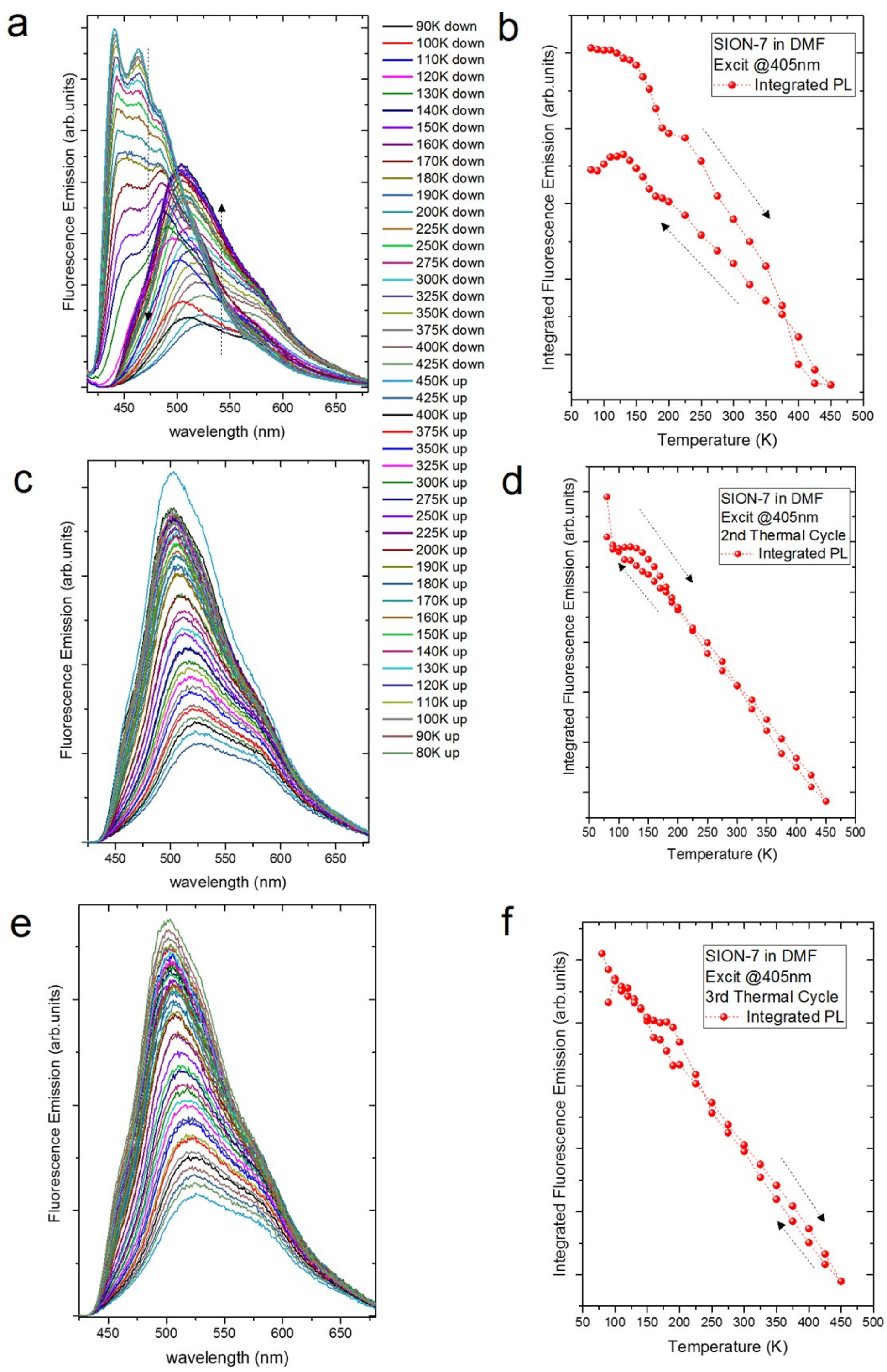

Fig. S12. Fluorescence spectra of **SION-7** measured in the 80–450 K temperature range on heating and cooling: (a) first thermal cycle, (c) second thermal cycle, (e) third thermal cycle. Integrated fluorescence intensity of **SION-7** in the 80–450 K temperature range on heating and cooling: (b) first thermal cycle, (d) second thermal cycle, (f) third thermal cycle.

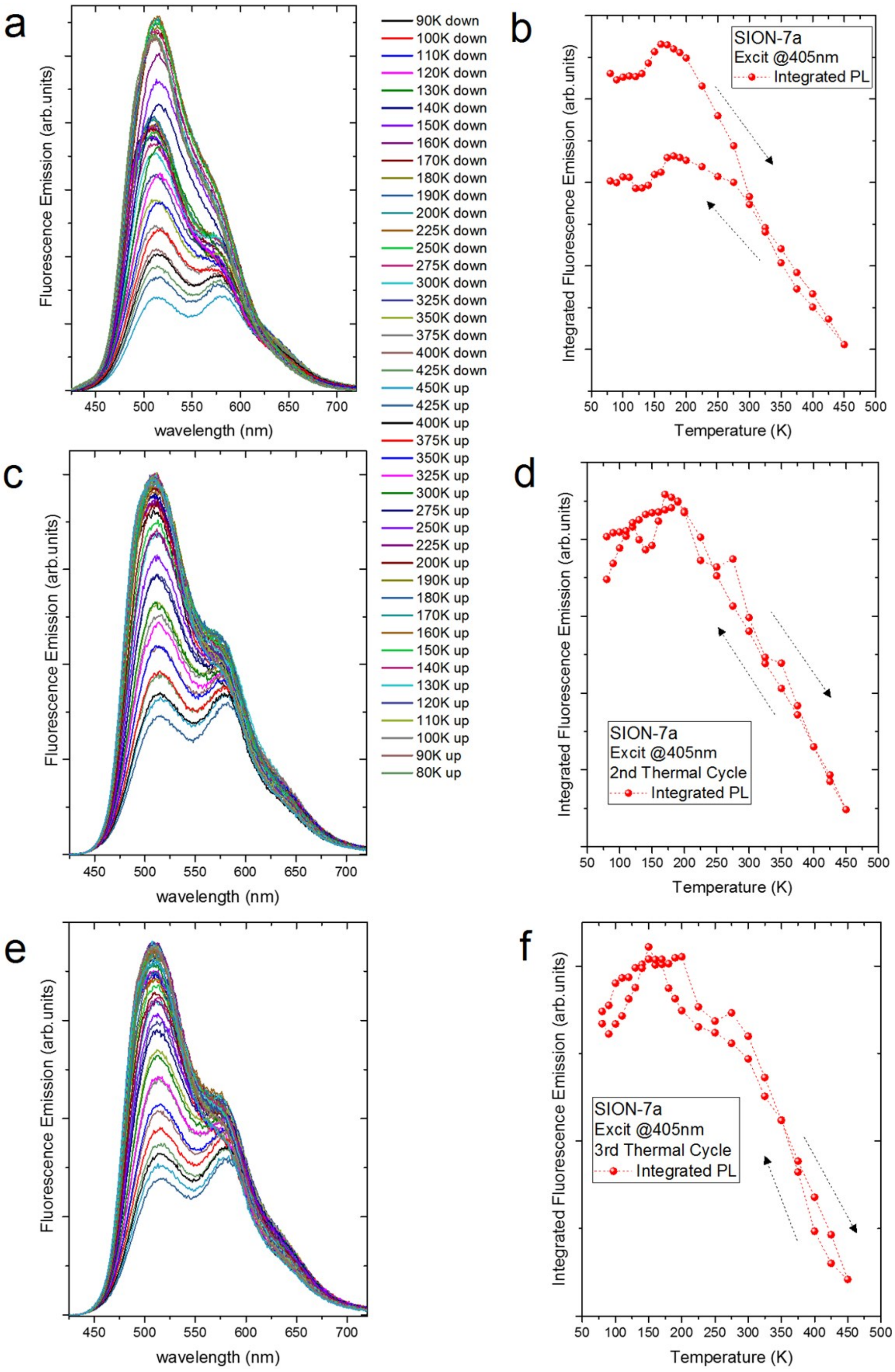

Fig. S13. Fluorescence spectra of **SION-7a** measured in the 80–450 K temperature range on heating and cooling: (a) first thermal cycle, (c) second thermal cycle, (e) third thermal cycle. Integrated fluorescence intensity of **SION-7a** in the 80–450 K temperature range on heating and cooling: (b) first thermal cycle, (d) second thermal cycle, (f) third thermal cycle.

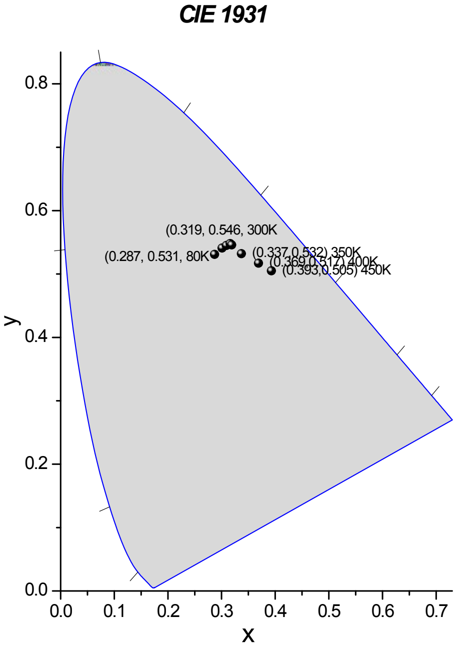

Fig. S14. CIE-1931 chromacity diagram of the fluorescence from **SION-7a** on heating in the 80–450 K temperature range.

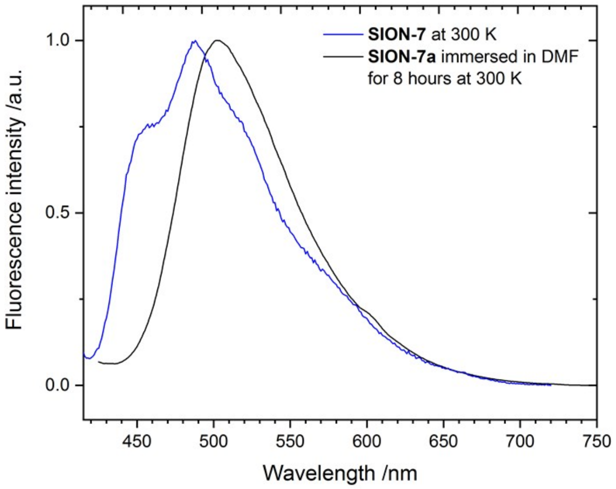

Fig. S15. Fluorescence spectrum of as-made **SION-7** measured at 300 K during the first heating cycle (blue plots) and that of the **SION-7a** immersed in DMF for 8 hours, filtered, dried in air and measured at 300 K (black plots). Lack of the low-wavelength feature in the second spectrum confirms that the monomer emission is not recovered.

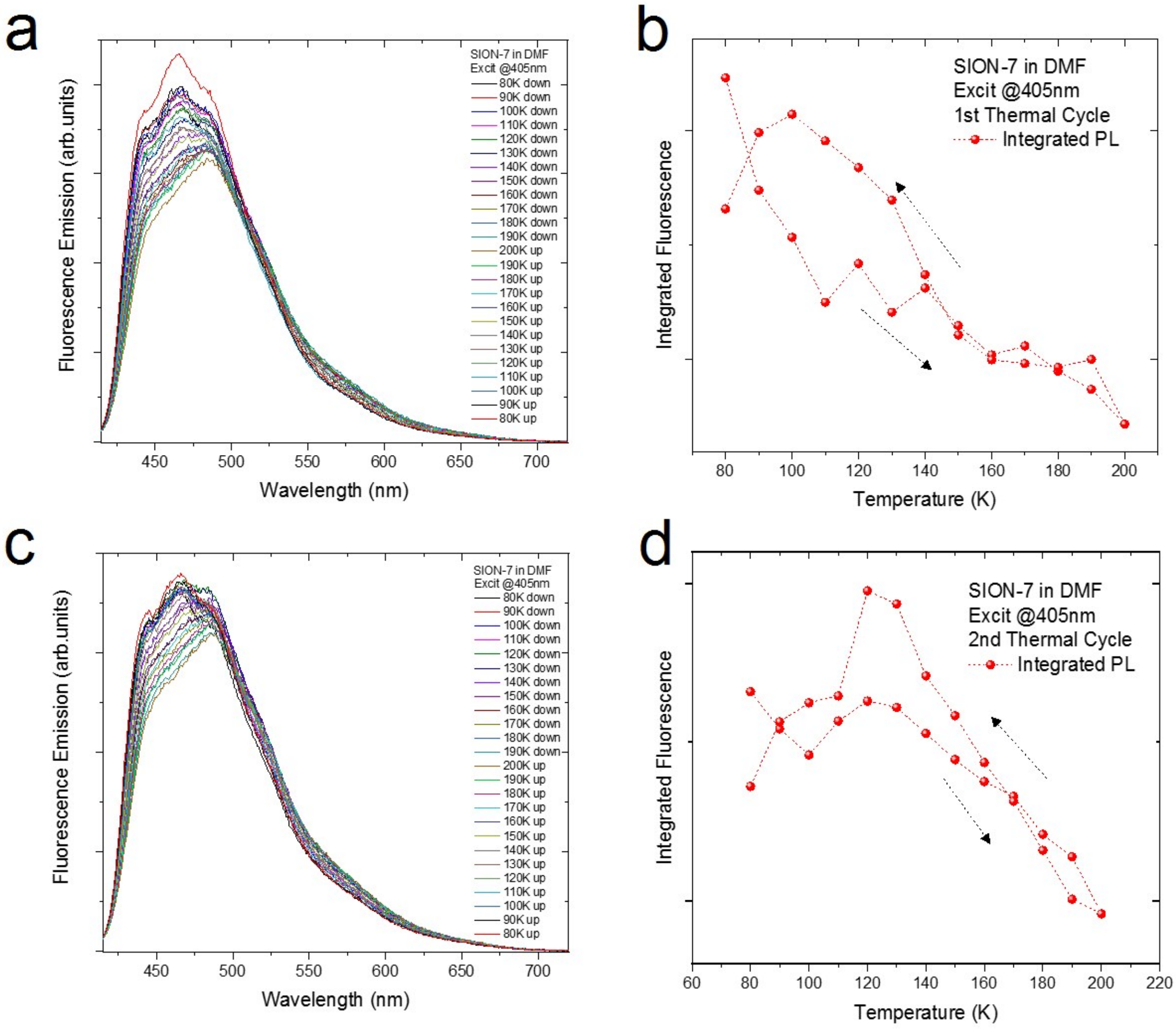

Fig. S16. Fluorescence spectra of **SION-7** measured in the 80–200 K temperature range on heating and cooling: (a) first thermal cycle, (c) second thermal cycle. Integrated fluorescence intensity of **SION-7** in the 80–200 K temperature range on heating and cooling: (b) first thermal cycle, (d) second thermal cycle.

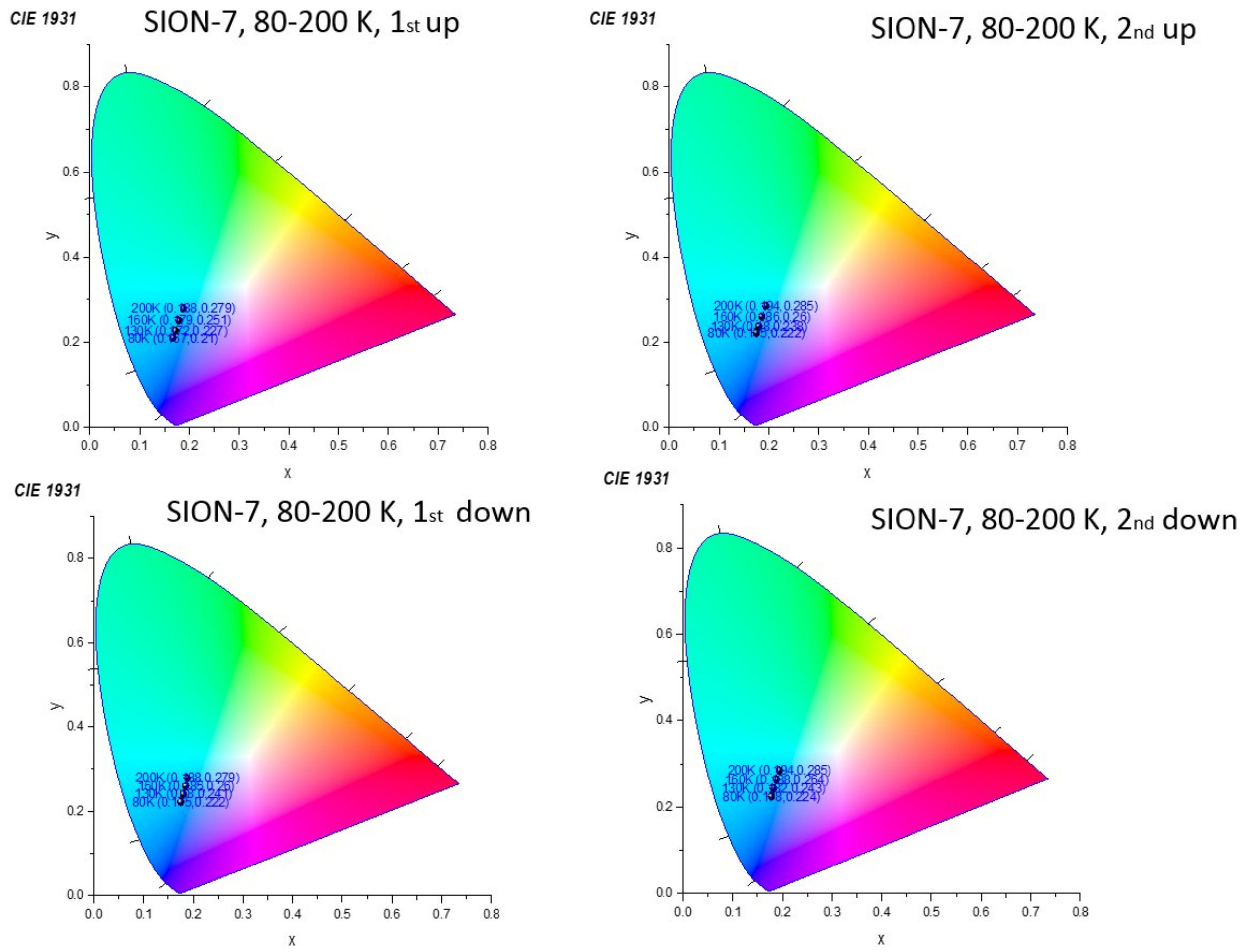

Fig. S17. CIE-1931 chromacity diagram of the fluorescence from **SION-7** on heating and cooling in the 80–200 K temperature range during the first and second thermal cycle.

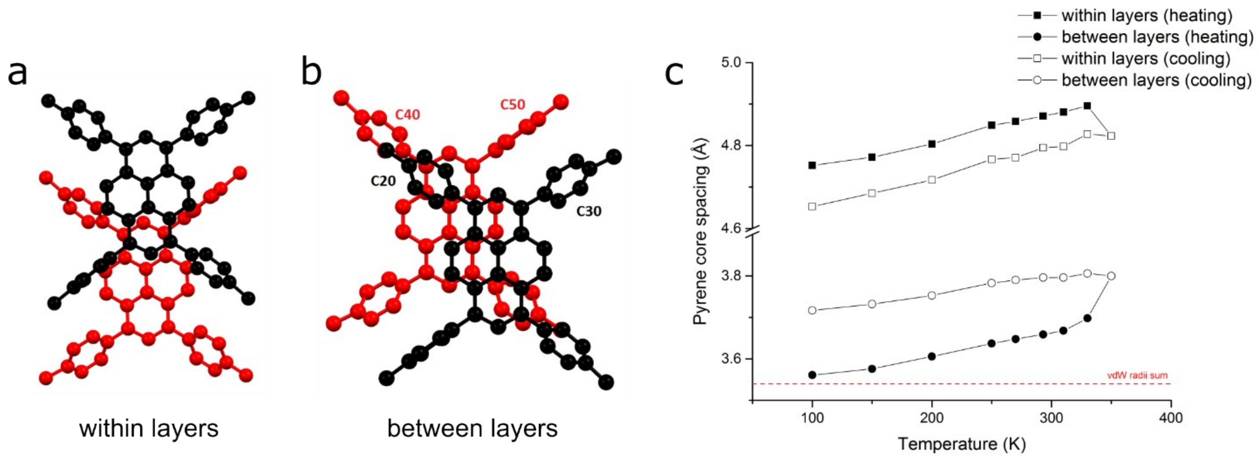

Fig. S18. A pair of $HTBAPy^{3-}$ ligands originating from (a) the same and (b) two neighbouring 2-dimensional layers of the ligands joined together by Mg_3-clusters. (c) Evolution of the pyrene core planes spacing with temperature.
